# High-Frequency and Low-Intensity Patterned Transcranial Magnetic Stimulation over Left Dorsolateral Prefrontal Cortex as Treatment for Major Depressive Disorder: A Report of 3 Cases

**DOI:** 10.1155/2021/5563017

**Published:** 2021-03-19

**Authors:** Lizbeth Castillo-Aguilar, Alma E. Ríos-Ponce, Edson Albano de Mendonca, Gabriel Villafuerte

**Affiliations:** ^1^Clínica Coyoacán, Mexico City, Mexico; ^2^Hospital Psiquiátrico “Dr. Samuel Ramírez Moreno”, State of Mexico, Mexico; ^3^Plan de Estudios Combinados en Medicina (PECEM), Facultad de Medicina, Universidad Nacional Autónoma de México, Mexico City, Mexico

## Abstract

Current transcranial magnetic stimulation devices apply intense (near 1 tesla) repetitive magnetic pulses over a specific area of the skull at relatively lower frequencies (1-50 Hz). Nevertheless, different studies have shown that very small magnetic fields, at higher frequencies (50-1000 Hz.), produce therapeutic effects in major depressive disorder. We report the application of high-frequency and low-intensity patterned magnetic pulses over the left prefrontal dorsolateral cortex in three subjects diagnosed with clinical major depressive disorder. All three patients showed sharp changes in their self-reports as well as in the standardized clinical assessment. Hypothesized mechanisms of action of this new variant of magnetic stimulation are discussed.

## 1. Introduction

Repetitive transcranial magnetic stimulation (rTMS) is a noninvasive neuromodulation technique that has shown to be effective for the treatment of major depressive disorder (MDD), especially in subjects in which pharmacological treatment has failed to improve depressive symptoms [1]. Current rTMS devices apply intense (near 1 tesla) repetitive magnetic pulses over a specific area of the skull at relatively lower frequencies (1-50 Hz). These rapidly changing and intense magnetic pulses produce their biological activity by inducing an electric current inside the brain [2]. Nevertheless, the threshold for pulsed magnetic field effects on biological systems has been estimated to be at much lower intensities (1 × 10^−7^ teslas) [3] and different studies have shown that very small magnetic fields, at higher frequencies (50-1000 Hz), do produce measurable changes in brain's activity [4] and even therapeutic effects in some nervous system pathologies [5, 6]. Rohan et al. published a study where they applied low-intensity magnetic fields (around 2 militeslas) at 1000 Hz producing an antidepressant effect in patients with bipolar depression [5]. However, this same protocol was used in patients with MDD having mixed results [7, 8]. Another protocol using similar parameters, applied with 7 coils to the whole brain was also used for the treatment of MDD with good results [9].

In this paper, we report the application of high-frequency and low-intensity patterned magnetic pulses with a circular coil of 60 mm of diameter over the F3 coordinate of the 10-20 EEG system (left prefrontal dorsolateral cortex) in three subjects diagnosed with clinical MDD. The coil used in this protocol was selected over a figure 8 coil as the intensities used in the present study are not able to produce a motor threshold; without a motor threshold and in order to assure that the prefrontal cortex was indeed stimulated, a circular coil with a larger area of stimulation was used.

**Figure 1:**
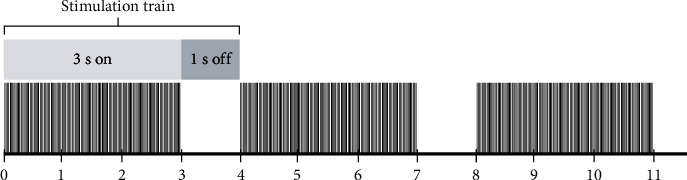
Stimulation pattern. The stimulation was divided in trains; each train consisted of a 3-second period of burst stimulation at 550-600 Hz and a 1-second period without stimulation. A total of 675 trains were applied during each session over the left prefrontal dorsolateral cortex.

The device used to apply the magnetic stimulation was designed and manufactured exclusively for this study by Actipulse Neuroscience (Boston, USA). The pulses were applied in trains: each train consisted of 3-second bursts of high-frequency pulses (550-600 Hz) alternated with 1 second without stimulation (see Figure 1 for more details about the stimulation pattern); a total of 675 trains (45 minutes of stimulation) were applied in each session. Each pulse had an approximate magnetic field intensity of 0.5 milliteslas. Sessions were applied to each patient once daily for 5 days each week, making a total of 15 sessions distributed in 3 weeks.

## 2. Case 1

### 2.1. Patient Information

Case 1 was a male, 69 years old, with Latin American ethnicity and with a family history of diabetes mellitus and colon cancer. The patient has a history of aortic valve calcification due to which he had to have an aortic valve surgery 5 years prior to this evaluation. During the aortic valve surgery, the patient suffered a cardiorespiratory arrest and, as consequence, he developed chronic posthypoxic myoclonus affecting his head, trunk, and superior limbs. After discharge, depressive symptoms started and were mainly associated with a feeling of worthlessness due to motor function impairment. Four years ago, the patient attended a psychiatric evaluation for the first time, referring depressed mood nearly every day, anhedonia, alexithymia, social isolation, insomnia, and anxiety symptoms, for which he was prescribed sertraline and clonazepam at unknown doses showing mild response.

### 2.2. Clinical Findings

The patient was conscious and oriented. He presented postural and action tremor in the upper limbs with an accentuation on the left side of the body, while on the lower limbs, he presented bradykinesia. The patient also presented gait changes including reduced stride length and speed, reduced arm movement, and deviation to the right side. At the time of assessment and treatment, the patient was taking sertraline, primidone, acenocumarol, clonazepam, metoprolol, paracetamol, losartan, and atorvastatin.

### 2.3. Diagnostic Assessment

The patient was assessed using the Montgomery–Åsberg Depression Rating Scale (MADRS), Beck Depression Inventory (BDI), Beck Anxiety Inventory (BAI), 12 item General Health Questionnaire (GHQ-12), Mini-Mental State Examination (MMSE), and the Athens Insomnia Scale (AIS). Moderate depression was found through the MADRS and the BDI with a score of 24 and 26 points, respectively, while on the BAI, severe anxiety was found (score of 32 points), as well as the presence of insomnia (score of 9). Cognition was preserved, demonstrated through the application of the MMSE (score of 28 points).

### 2.4. Follow-Up and Outcomes

All tests were reassessed after 15 sessions of HFLI TMS, and an improvement in all measures was observed. On the other hand, both the MADRS (score of 10 points) and the BDI (score of 13 points) reduced their scores, indicating a change from moderate to mild depression, as well as the BAI, which indicated the presence of moderate anxiety (score of 23 points). Meanwhile, insomnia (AIS = 6) and cognition scores (MMSE = 30) were also improved, returning to normal values. In the self-report, the patient reported a clear improvement in mood, anxiety, and sleep disturbances.

## 3. Case 2

### 3.1. Patient Information

Case 2 was a female, 27 years old, with Latin American ethnicity and with a family history of cardiac disease, asthma, diabetes mellitus, and pulmonary emphysema. She was diagnosed with attention deficit and hyperactivity disorder 10 years prior to the evaluation; 9 years prior, a hygroma was found incidentally in an MRI scan performed for other reasons. She has a positive history of tobacco and drug use including cannabis, cocaine, LSD, methamphetamine, ecstasy, and hallucinogens. The onset of psychiatric symptoms was at age 12 with anhedonia, social isolation, apathy, emotional liability, and sleeping problems; at the age of 18, she had a suicide attempt and was institutionalized for a month. Trials with different medications (fluoxetine, sertraline, carbamazepine, valproate, and clonazepam) had a poor effect in remission of depressive symptoms and complete remission was never achieved.

### 3.2. Clinical Findings

The patient was conscious and oriented. She presents with anxiety-related tachycardia, as well as excessive sweat and paresthesia. At the time of assessment and treatment, the patient was taking a stable dose of venlafaxine for over 3 months.

### 3.3. Diagnostic Assessment

The patient was assessed with the same scales and inventories as the first case. On depression tests, the patient presented moderate depression through the MADRS (score of 30 points), while on the BDI, she presented severe depression (score of 46 points). On the other hand, severe anxiety was found (BAI score of 37 points), as well as the presence of insomnia (score of 21 points in AIS). Cognition was fully preserved, demonstrated through a flawless MMSE score (30 points).

### 3.4. Follow-Up and Outcomes

Tests were reassessed after 15 sessions of HFLI TMS, and an improvement in all measures was observed, reaching minimum levels. Both the MADRS (score of 5 points) and the BDI (score of 0 points) reduced their scores, indicating the absence or minimum presence of depressive symptoms. While the BAI indicated a minimum presence of anxiety (score of 7 points) and the AIS (score of 6 points) showed an absence of insomnia symptoms. Finally, the cognitive score was decreased by two points (MMSE = 28 points); however, it remained within normal values. The self-report of the patient corroborated the reported clinimetric changes; the patient reported minimum depressive, anxiety, and insomnia symptoms.

## 4. Case 3

### 4.1. Patient Information

Case was a 38-year-old Latin American female with a family history of cardiac disease, arterial hypertension, pulmonary, and testicular cancer. The patient was diagnosed 2 years prior to evaluation with borderline personality disorder and had positive tobacco and alcohol use, reaching inebriation at least once every 15 days. The patient presented depressive symptoms with labile mood for the first time at 18 years of age. At 21 years old, she was diagnosed with postpartum depression after symptoms of isolation, anhedonia, and emotional lability augmented. She had two suicide attempts at age 24 and 28, both of which were followed by the hospitalization of the patient. Since age 24, she had received several antidepressants intermittently (mainly fluoxetine and sertraline), with poor improvement of symptoms. Two months ago, depressive symptoms increased, and she started fluoxetine 40 mg daily by herself. Poor symptomatic response was achieved, and she continued with anhedonia, hopelessness, sleeping problems, irritability, and anxiety.

### 4.2. Clinical Findings

The patient was conscious and oriented. She complained of occasional tachycardia and lower limb paresthesia while being stressed, as well as acid reflux with every meal, leading to a diminishment in daily food intake; additionally, the patient appears to be sleepy and tired. At the time of assessment and treatment, the patient had suspended medication without physician supervision.

### 4.3. Diagnostic Assessment

The patient was assessed with the same scales and inventories as in previous cases. The MADRS showed moderate depression (score of 28 points), and the BDI indicated the presence of severe depression (score of 42 points). Meanwhile, the BAI indicated the presence of severe anxiety (score of 37 points), as well as the presence of insomnia (score of 10 in AIS). Finally, the MMSE indicated no impairment; however, the score is on a limit cut-off value (score of 24 points).

### 4.4. Follow-Up and Outcomes

Reassessment was performed after 15 sessions of HFLI TMS, showing an improvement in all measures. The BDI (score of 9 points) demonstrated a reduction of depressive symptoms, reaching minimum levels of depression, while the MADRS (score of 10 points) score reduction reached mild depression levels. The BAI also indicated a minimum presence of anxiety (score of 5 points), and the AIS (score of 7 points) showed a minimum presence of insomnia symptoms. Finally, the cognitive score improved by five points (MMSE = 29 points), which could indicate that baseline evaluation could be influenced by concurrent MDD. The changes in the scales were corroborated by the self-report of the patient.

**Figure 2:**
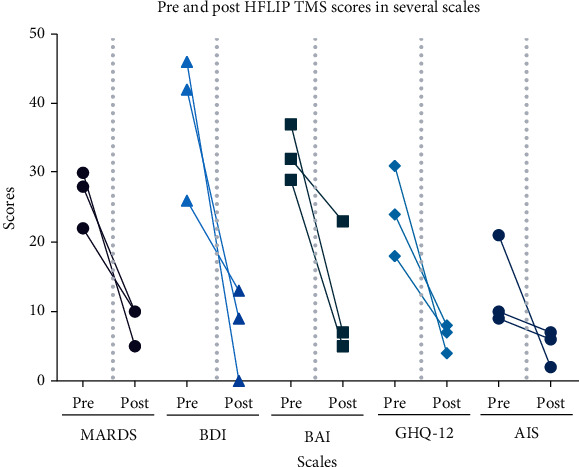
Pre- and post-HFLIP TMS scores in several scales. Several clinimetric scores were performed before and after HFLIP TMS: Montgomery–Åsberg Depression Rating Scale (MADRS), Beck Depression Inventory (BDI), Beck Anxiety Inventory (BAI), 12 item General Health Questionnaire (GHQ-12), Mini-Mental State Examination (MMSE), and the Athens Insomnia Scale (AIS). All three subjects showed improvement in all measured scales.

Before and after changes in scales for the three subjects are presented in Figure 2.

## 5. Discussion

In this report, three patients with different history and clinical presentation of MDD were treated with high-frequency and low-intensity magnetic patterned pulses over the left dorsolateral prefrontal cortex and showed remarkable clinical improvement after 15 sessions of stimulation.

While each patient presented a different clinical background, all three patients showed sharp changes in their self-report and in the standardized clinical assessments. The mechanisms responsible for the observed clinical changes in these patients are almost certainly different from those produced by classical TMS devices. The pulse intensity applied by this device is several orders of magnitude lower than the one required to generate a motor evoked potential, so direct depolarization of neurons does not seem like a viable mechanism of the observed antidepressant effect [10]. Even if there is no direct depolarization of neurons, magnetic pulses at a low subthreshold intensity and relatively high frequency have demonstrated to change cortical excitability [11], modify brain metabolism [12], and change neurocognitive function in humans [13]. How does these kinds of magnetic fields modify the brain's activity is not completely understood, but animal and human evidence have shown an increase in plasticity [14], BDNF [15], and an anti-inflammatory effect [16], which, coincidentally, are normally affected in MDD [10].

Other studies using magnetic pulses have reported mixed results in the antidepressant effects of magnetic stimulation in a similar window of frequencies and intensities. Rohan et al. first published that the application of magnetic pulses at 1000 Hz and an intensity of no more than 2 milliteslas to the whole brain reduced depressive symptoms in patients with bipolar disorder and MDD compared to sham stimulation with just one session of stimulation [5]. Years after, a double-blind proof of concept clinical trial showed no difference between sham and real stimulation with this same stimulation protocol and device in improving depression scores in subjects with unipolar MDD, leading to the conclusion that more sessions of stimulation and longer exposure time could explain the lack of efficacy of this trial [7]. A new and more recent double-blind clinical trial using this same stimulation protocol showed improvement in mood scores in real stimulation compared with sham with three sessions of stimulation [8].

Taking into count those previous studies, we designed a stimulation protocol that acknowledged three main points from previously reported protocols.

Firstly, as classical rTMS devices, we decided to focus the stimulation just in one area of the brain instead of applying a diffuse and global magnetic field to the whole skull. Pathophysiologically, we considered it important to focus the magnetic stimulation on one area known to be affected in MDD such as the left prefrontal dorsolateral cortex [17]. Secondly, the pattern of stimulation seems to be very important in determining the effects of both classical [18] and low-intensity magnetic stimulation [19]. That is why we chose a novel pattern of stimulation that has been shown to modify the brain's activity in both animal models (unpublished data) and humans. This novel stimulation pattern has shown to improve mood and insomnia symptoms in healthy young people [20].

Lastly, while neuroplastic changes can occur after just one session of rTMS [18], lasting and clinically relevant changes typically occur after at least 10 sessions of classical rTMS devices [21]; we hypothesized that applying a similar number of sessions as classical rTMS stimulation could lead to a more pronounced and consistent antidepressant effect compared to other low-intensity protocols.

We advise to regard this report with caution, as only three cases without proper controls are described, so placebo effects could not be evaluated. Also, the size of the group and its heterogeneity could have influenced the results obtained in this work, as patients were very different amongst themselves.

To reach stronger conclusions about the effect of HFLIP TMS, the group size must be augmented, and their heterogeneity reduced by the application of strict selection criteria, rather than a sample selected by convenience. Moving forward, clinical trials using this new HFLIP TMS protocol should be performed with appropriate sham control to correctly assess the clinical efficacy, as well as to clarify if the placebo effect could play a role in the improvement seen on the patients or other effects this technique could generate in MDD subjects.

## Data Availability

Anonymized data of the applied scores are available upon request.
